# High Thermal Conductivity of Flake Graphite Reinforced Polyethylene Composites Fabricated by the Powder Mixing Method and the Melt-Extruding Process

**DOI:** 10.3390/polym10070693

**Published:** 2018-06-21

**Authors:** Zhichun Liu, Runchun Tu, Quanwen Liao, Hanlin Hu, Jinguo Yang, Yan He, Huiguang Bian, Lianxiang Ma, Wei Liu

**Affiliations:** 1 School of Energy and Power Engineering, Huazhong University of Science and Technology (HUST), Wuhan 430074, China; Turunchun@hust.edu.cn (R.T.); Liaoquanwen@hust.edu.cn (Q.L.); Huhanlin@hust.edu.cn (H.H.); jg_yang@hust.edu.cn (J.Y.); 2 College of Electromechanical Engineering, Qingdao University of Science & Technology, Qingdao 266061, China; heyan_sd@163.com (Y.H.); bianhuiguang@163.com (H.B.); oldhorse@qust.edu.cn (L.M.)

**Keywords:** polyethylene, flake graphite, composites, synergistic effect, thermal conductivity

## Abstract

The thermal conductivity of flake graphite (FG) particulates reinforced high density polyethylene (HDPE) composites was systematically investigated under a special dispersion state of FG particles. The effects of particle size, weight filling ratio and proportion of various sizes were discussed in detail. A special composite (15 wt % 500 μm/10 wt % 200 μm/10 wt % 20 μm/5 wt % 2 μm FG + 60 wt % polyethylene (PE)) with a high thermal conductivity about 2.49 W/(m·K) was produced by combining the synergistic effect of several fillers. The component material size distribution was employed to analyze the effect of particle size. And scanning electron microscope (SEM) was adopted to observe the FG network in the composites. Thermogravimetric analysis (TGA) revealed the good thermal stability of composites. Differential scanning calorimetry (DSC) indicated that all composites own a similar melting temperature. Sample compression experiment indicated that all composites still exhibit high mechanical strength. Consequently, the easy-making flake graphite reinforced polyethylene composites with a high thermal conductivity would have a wide application in the new material field, such as a thermal interface material, a heat exchanger, voltage cable, etc.

## 1. Introduction

Thermally conducting, polymer-matrix composites are increasingly important for electronic packaging, polymer heat exchangers, phase-change materials and aerospace engineering. Therefore, significant efforts have been made toward improving the thermal conductivity of thermally conducting composites [1,2]. In fact, there are two main solutions to achieve this target. On the one hand, enhancing the thermal conduction based on polymer matrix [3,4,5], including making the alignment of polymer chains, increasing the rigidity of a molecular bond and improving the interchain interactions. In these fields, molecular dynamics simulation method is a useful tool to predict the thermal properties of polymer [6,7,8,9], which exhibits good accuracy and easy-operating property. Moreover, the molecular dynamics simulation method could build a fundamental linkage between molecular characteristics and macroscopic thermal properties [10,11]. On the other hand, it could also improve the thermal conductive capability of polymers by doping high thermal conductivity fillers, such as carbon nanotubes, graphene, graphite nanoplatelets, ceramics and metals [12,13,14,15,16,17,18]. This work plays an important role in polymer thermal engineering as it is easy to be realized.

Thermally conductive polymeric composites are usually designed by blending polymeric matrix with economic and convenient fillers, because the fabricating cost is low. Traditional fillers include copper powders, nickel powders, aluminium powders, metal alloy and carbon materials. Nowadays, carbon materials have got increasing attention as they are equipped with superior properties, such as light weight, high strength, strong acid resistance, large specific surface area and high thermal conductivity. In the carbon family, flake graphite (FG) is a common branch, which could be easily found in nature. And FG is widely utilized in thermal conducting materials as it has good plasticity, high thermal conductivity and excellent high melting point. It is highly stable even in severe conditions such as acidic and alkaline solutions [19]. For polymer matrix, polyethylene (PE) is a widely-used one in polymer family. In fact, PE owns relatively higher thermal conductivity (0.2~0.4 W/(m·K)) than other polymers.

Nowadays, there have been two interesting and advanced studies toward PE [20,21]. One is the nanoporous metallized PE textile made by Cui et al. [20], which could warm up human body by fully utilizing the different radiation rate of carbon and copper. Another is the scalable-manufactured randomized glass-polymer hybrid metamaterial for daytime radiative cooling, which is designed by Yang’s group [21]. These two original materials both make full use of PE as it is environmental and easy to process. So it is easy to put forward that we may obtain high thermally conducting PE/FG composites by combining each component. Many studies have focused on graphite/polymer composites [19,22,23,24]. Ye et al. [19] studied the thermal conductivity of high density polyethylene filled with colloid graphite and home-made expanded graphite and they found that graphite is a kind of ideal thermal conductive agent as it has large specific surface area. However, they just thought about fillers with one size. Zhou et al. [22] prepared oriented graphite/polymer composite sheets with high thermal conductivity by the tape casting method. Zhang et al. [14] introduced the improved thermal property of a multilayered graphite nanoplatelets filled silicone composites. They highlighted the advantage of the multilayered graphite nanoplatelets in thermal conduction as they have high aspect ratio. Since the interface thermal resistance existing between matrix and fillers hinders the thermal transport, some studies focused on the covalent and non-covalent functional fillers to improve the whole thermal conductivity [11,12]. However, this method is hard for volume production due to the high cost. Therefore, investigating the impact of the wide particle size distribution is still an interesting work to carry on.

**Figure 1:**
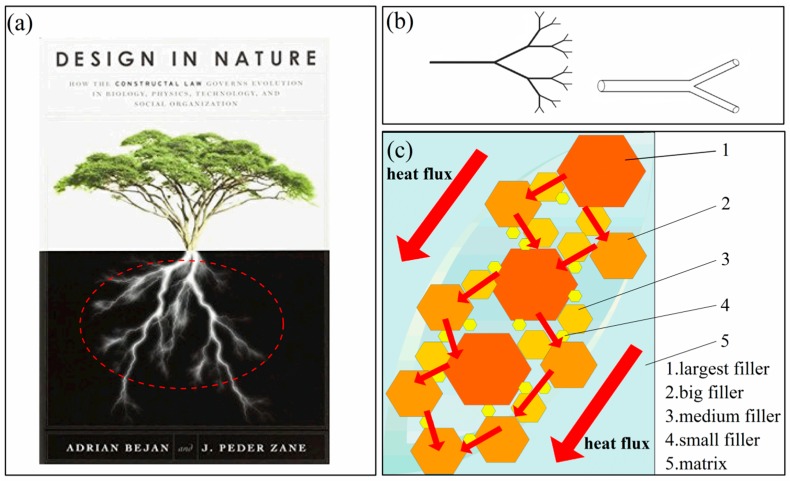
(**a**) A fractal root system of the tree [26]; (**b**) a classical constructal network; and (**c**) diagram of several-sized fillers filling in the matrix (2 dimensions).

Zhou et al. [17,25] put forward that the combined use of hybrid fillers obtains a higher thermal conductivity compared to one kind of filler. In recent years, Kim et al. [13] found that the thermal conductivity of the polycarbonate composite filled with both 9.9 wt % expanded graphite and 0.1 wt % multi-walled carbon nanotube fillers was synergistically improved by 49% compared to the polycarbonate (PC) composite filled with 10 wt % expanded graphite (EG) alone. Therefore, synergetic doping for PE/FG composites may have more research values to improve the thermal conductivity. Just like a fractal root system of the tree shown in Figure 1a, which helps the tree to absorb nutrients and water from the soil in a perfect way. In past studies, Adrian Bejan introduced the constructal-theory network of conducting paths for cooling a heat generating volume [26], one classical fractal network is shown in Figure 1b. Therefore, what is the impact of several-size fillers on the thermal conductivity of PE composites? For composites, large fillers are just like backbones while the small fillers represent branches. Could it form a better thermal network for heat transfer just like what is shown in Figure 1c? It is an important and interesting research.

One classical way to construct a heat conductive path is mixing filler particles and polymer particles together, then melt-extruding the mixture to get a special filler dispersion state in which the polymer particles are surrounded by heat conductive fillers [27,28,29]. In this paper, high density polyethylene (HDPE)/FG composites were fabricated by an easy-making powder mixing method and melt-extruding process. Particularly, the impact of the wide-ranging size and filling ratio of FG particles on the thermal conductivity of composites were systematically studied in detail. For some typical composites, we made specific thermal and mechanical analysis to evaluate their overall quality. These abundant and precious experimental results would help to guide the industrial production of PE/FG composites with a high thermal conductivity.

## 2. Experimental Methods

### 2.1. Materials and Morphology

The matrix here was high density polyethylene (HDPE), supplied by the Qilu Petroleum Corporation, Zibo, China. The HDPE has a melting flow index (MFI) of 0.35 g/10 min, a melting point of 423 K and a density of 0.95 g/cm^3^. The dominating filler was FG, which has a melting temperature of 3273 K, and the average proportion of carbon element is over 99 wt %, purchased from original Shandong South Villa Graphite Ore Factory, Qingdao, China. There are four different sizes of FG, including 500, 200, 20 and 2 μm, which is marked by the manufacturer. The density of 500, 200 and 20 μm FG is around 1.42 g/cm^3^, while the density of 2 μm FG is around 0.35 g/cm^3^. Other supplementary fillers were multiwalled carbon nanotube (MWCNTs) and high quality graphene. The MWCNTs was obtained from Zheda Quality, Hangzhou, China, with a length ranging from 10–20 μm and specific surface area around 260–560 m^2^/g, the purity is over 94%. And the high quality graphene (99.9%) was provided by Hefei Microcrystalline Materials Technology Co. LTD, Hefei, China, whose specific surface area is 100–150 m^2^/g and the average particle size is around 5–10 μm. The average diameter of MWCNTs is around 12–25 nm, and the thickness of graphene is less than 10 nm. The average particle size of materials measured by laser particle size distribution instrument (MS2000, supplied by Malvern, England) is shown in Table 1. It is indicated that the test size is in good agreement with the factory size, which exhibits as normal distributions. Here, all materials are seen as spherical during the testing process.

### 2.2. Experiment and Measurement

**Figure 2:**
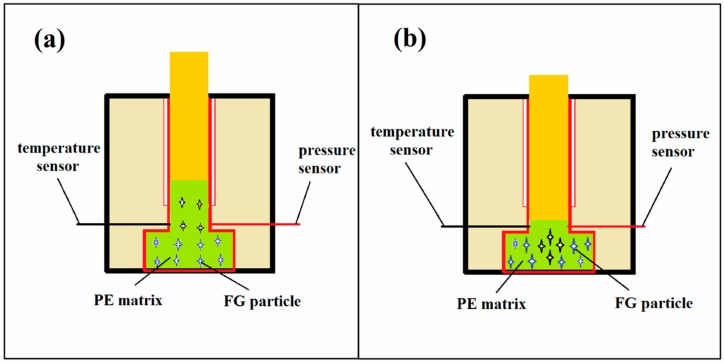
Schematic diagram of the molding process using twice melting and extruding method: (**a**) extrude the solid powders and heat at 523 K for 60 min and (**b**) press the molten mixtures at 20 MPa and then heat at 423 K for 30 min.

In this paper, an easy-making powder mixing method and melt-extruding process were employed to prepare the composites by compounding HDPE with graphite powders etc., and the molding process is shown in Figure 2. The mixing process was operated on the vortex vibrating instrument (Lianlink JHX28H, Beijing, China) and the blending time was around 20 min. The mixtures have been fabricated through the twice pressing process. That is firstly, extruding the mixing powders to exhaust the air as much as possible and heat at 523 K for 60 min; secondly, pressing the molten mixtures at 20 MPa and then keep the temperature at 423 K for 20 min. After washing for 3 min to cool down the sample, the mixture were produced in a 40 mm × 20 mm × 4 mm plate. Thus, HDPE/FG composites with graphite powders ranging from 0 to 40 wt % have been well prepared. In order to better investigate the influence of the filler itself on the thermal conductivity, we keep the same preparing condition to reduce the uncertainties in preparing process.

After molding, the thermal conductivity of HDPE/FG specimens is measured by hot desk method (TC-3000, XIATECH, Xi’an, China) at room temperature. To keep the accuracy of the thermal conductivity, we made an average of 6 measured data to get average value of thermal conductivity. In order to observe the distribution of fillers in PE matrix, the microstructure of the composites was observed by scanning electron microscope (SEM, ZEISS, Sigma, Neustadt, Germany) instrument with an acceleration voltage of 15 kV. In order to further investigate the thermal behavior of some typical composites, a slight fraction of the composites was measured by thermogravimetric analysis (TGA, STA 449F3, NETZSCH Company, Ahlden, Germany) and the heat was measured through differential scanning calorimetry (DSC, STA 449F3, NETZSCH Company, Ahlden, Germany) at the heating rate of 15 K/min. Finally, classical compress experiments (YES-2000, Zhongte Testing Machine Company, Jinan, China) were carried out to evaluate the mechanical property of these composites. Samples with a size of 10 mm × 10 mm × 4 mm were gradually compressed under a press until broken down, the compressing rate was 3 min/mm and the detailed stress-strain curves of the compression process were analyzed. The uncertainties mainly come from the sample flatness.

## 3. Results and Discussion

### 3.1. Influence of the Weight Content of one Size of FG on the Thermal Conductivity of HDPE/FG

**Figure 3:**
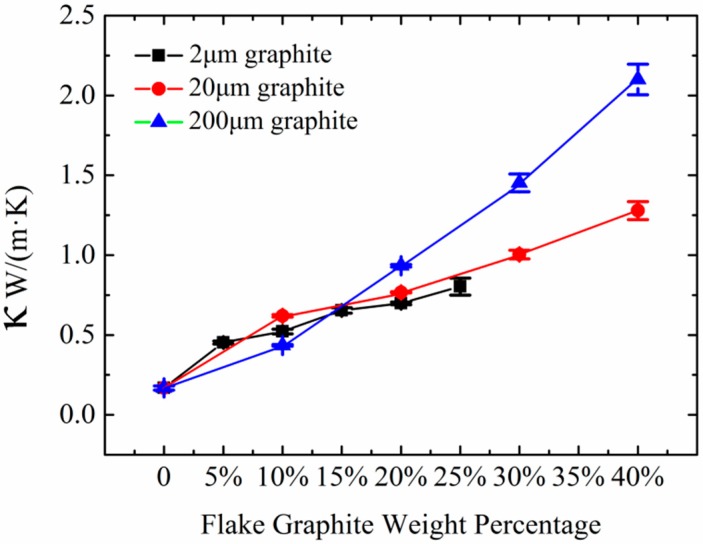
Thermal conductivities of the HDPE/FG composites versus FG weight percentage.

The propagating rate of the thermal flow through a nonmetallic solid depends on the coupling intensity of the vibration movements of the atoms and groups of adjacent atoms. As the backbone of PE chains is carbon element, which is the component element of FG, there should be some similar vibration movements in these two substances [30]. And the homogeneous FG has a large contact area with HDPE, facilitating heat flow and promoting phonon diffusion in the composites, so how about the thermal conductivity of HDPE/FG composites? Thermal conductivity of HDPE filled with various weight filling ratio of FG is indicated in Figure 3. There are three typical particle sizes, including 2, 20 and 200 μm. An obvious difference between 2 μm FG and 20 μm FG is the density. The former size is lighter as it has much bigger specific surface area. 200 μm FG has a more visible flaky shape than 20 μm FG. Results showed that with the increase of the FG content, the thermal conductivity of all HDPE/FG composites increased. In order to maintain the quality and strength of composites, the highest filling ratio of 2 μm FG is less than 25 wt % while the content of 20 μm FG and 200 μm FG could increase to 40 wt %.

Here, filler size plays a dominant role in the thermal conductivity. As shown in Figure 3, composites consisted of small fillers exhibit better thermal conduction when the filling ratio is under 15 wt %. When the FG content is over 15 wt %, composites with 200 μm FG have a higher thermal conductivity than other two fillers. Particularly, the thermal conductivity of the composite could achieve 2.16 W/(m·K) when the content of 200 μm FG was 40 wt %, which was higher than the composite consisted of 20 μm FG, whose thermal conductivity was 1.32 W/(m·K). According to these results, it could be easily speculated that small fillers exhibit better dispersibility in HDPE matrix when the filling content is low. When the filling ratio reaches 40 wt %, the composite with large FG particles shows better thermal conducting ability as the thermal network of graphite flake is well formed. Small FG particles also form network, but more thermal contact resistance also exists between one FG particle and another. Composites with small filler particles have large interface area causing phonon scattering and hindering phonon transport, and thus lead to a lower thermal conductivity [31]. Wu et al. [32] also studied the thermal conductivity of graphite nanoplatelets/polyetherimide (PEI) system with different filler sizes from 1 to 15 μm. They also found the similar phenomenon. Therefore, there may exist a balance between dispersion state and thermal contact resistance for the PE/FG composites. And a better thermal network should exist in the composites.

### 3.2. Thermal Conductivities of HDPE with 5 wt % Fillers

**Figure 4:**
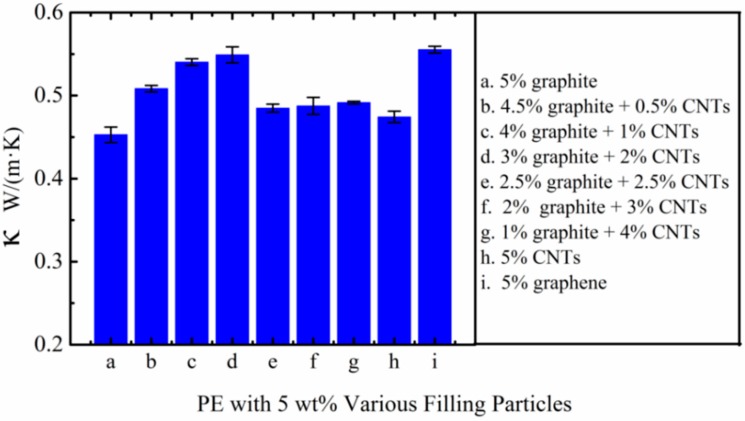
Thermal conductivities of HDPE with various 5 wt % fillers (the average size of FG is 2 μm).

In order to investigate the relationship between the thermal conductivity of composites and the filling material under a low filling ratio (5 wt %), we made a comparison about the thermal conductivities between several composites. The thermal conductivities are indicated in Figure 4. Results showed that the thermal conductivities of all samples with 5 wt % fillers are 0.45~0.55 W/(m·K). Being limited to the filling content, thermal conductivities are not apparently different between these fillers. The thermal conductivity of the composite filled with 5 wt % 2 μm FG is 0.45 W/ (m·K) and the sample filled with 5 wt % CNTs is 0.48 W/(m·K). The highest thermal conductivity of the sample filled with graphene is 0.55 W/(m·K). The limited thermal conductivity of these composites filled with nanofillers is mainly caused by the slight aggregation and defects of nanofillers [33]. And the consequent formation of cracks, pores, or pin holes in composites decreases their properties compared to expectations. The interesting finding is that by combining micron material and CNTs or graphene could achieve a higher thermal conductivity, such as the sample filled with 3 wt % 2 μm FG and 2 wt % MWCNTs [0.55 W/(m·K)]. Hence, it is of great significance to make a full use of the synergetic effect of several materials to achieve a higher thermal conductivity.

### 3.3. Influence of Hybrid Filling Mode on the Thermal Conductivities of HDPE with 40 wt % Fillers

**Figure 5:**
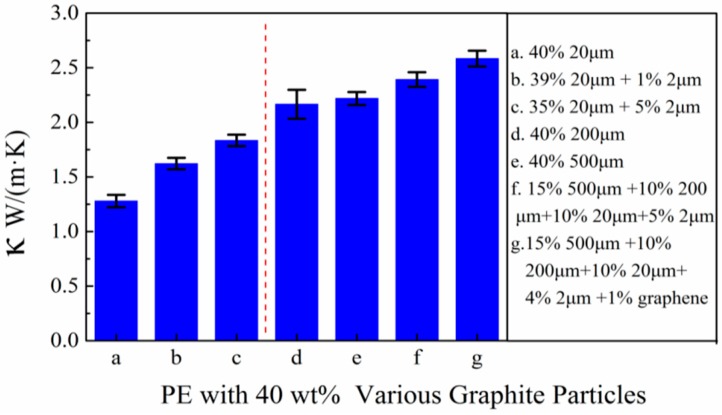
Thermal conductivities of HDPE with 40 wt % various filling particles (the size unit is μm).

Based on above results, combined filling method shows a positive impact on improving the thermal conductivity of composites. For industrial application, the thermal conductive polymer materials equipped with a thermal conductivity around 1~30 W/(m·K) are necessary. However, the HDPE/FG composite could not fulfill the requirement with slight FG fillers. To achieve the required thermal conductivity, various filling patterns of 40 wt % filling ratio are systematically studied in this part, results are shown in Figure 5. It was shown that under high FG filling content, composites with several fillers exhibit an advantage in thermal conduction over composites with just one filler. The similar phenomenon could be found in small fillers (20 or 2 μm) and large fillers (500 or 200 μm etc.). For example, the composite consisted of HDPE and 35 wt % 20 μm plus 5 wt % 2 μm FG could achieve a thermal conductivity of 1.83 W/(m·K) while the thermal conductivity of HDPE with 40 wt % 20 μm FG is only 1.28 W/(m·K). Furthermore, the highlight is that the composite consisted of 4 kinds of FG (15 wt % 500 μm + 10 wt % 200 μm + 10 wt % 20 μm + 5 wt % 2 μm) could achieve a thermal conductivity as high as 2.49 W/(m·K). The improvement is mainly derived from two aspects. One is abundant FG particles with large size like 200 or 500 μm have an advantage in forming a thermal conductive network. Another reason is that a little small FG particles optimize the establishment of the thermal conductive network by filling the vacancies between large FG particles. To further improve the thermal conductivity, 1 wt % graphene is added to replace 1 wt % 2 μm graphene, then the highest thermal conductivity [2.65 W/(m·K)] is achieved in the experiment, which is comparable to PA6/PC/graphite composites made by Zhou’s group [24].

At the same time, comparing the pattern a with pattern d or e in Figure 5, we can conclude that composites with large FG particles show better thermally conductive ability than small particles under 40 wt % filling ratio. The reasonable explanation for this phenomenon is consisted of two aspects. The first reason is that under 40 wt % filling ratio, both large FG particles and small FG particles are able to form thermal network by contacting with each other, but the thermal contact resistance of the former is smaller. More contacting points exist in small FG particles, which is harmful to the whole thermal conduction. Secondly, large FG particles could be more easily to form superior thermal network as it is harder to be removed during our melting and extruding process. This phenomenon was often overlooked in past studies [15,26]. However, large particles may have problem in contacting with each other, so by adding proper small particles, such as 2 μm FG or graphene, this problem can be alleviated to some extent. By combining above factors, a HDPE/FG composite with relatively better thermal conducting property is produced after doping.

### 3.4. Morphology of Composites

**Figure 6:**
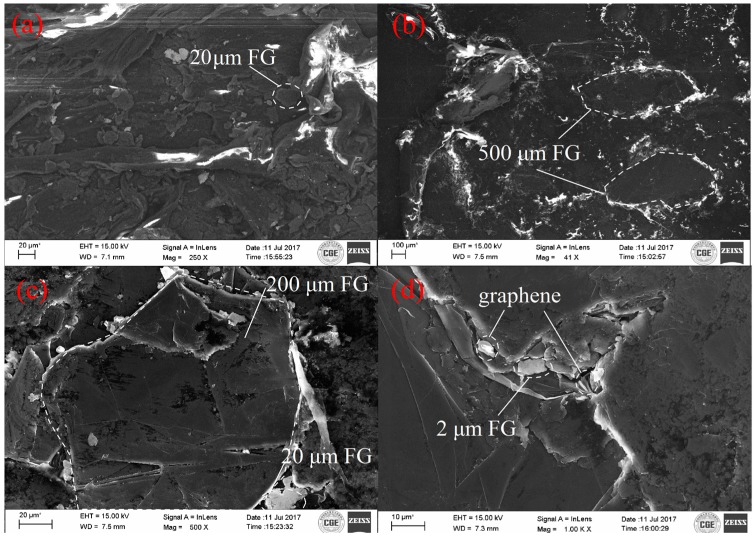
The scanning electron microscope (SEM) micrographs of HDPE/FG composites; (**a**) 40 wt % 20 μm FG and 60 wt % HDPE; (**b**) 40 wt % 500 μm FG and 60 wt % HDPE; (**c**) 15 wt %, 500 μm/10 wt % 200 μm/10 wt % 20 μm/5 wt % 2 μm FG and 60 wt % HDPE; (**d**) 15 wt % 500 μm/10 wt % 200 μm/10 wt % 20 μm/4 wt % 2 μm FG/1 wt % graphene and 60 wt % HDPE.

For better observation of the dispersion of FG, the SEM sample was prepared. The morphology of the obtained HDPE/FG composites (arbitrarily chosen with a composition of 60 wt %/40 wt %) at various filling patterns is shown in Figure 6. According to Figure 6a,b, both small FG and large FG have been dispersed and molded well in the composites using twice melt-extruding powders method. By utilizing this easily-operated method, we can take full advantage of the viscosity of the melting HDPE. Additionally, it is shown in Figure 6c,d that small particles like 20 μm FG, 2 μm FG or graphene have filled the vacancy in the interface between several large particles. The SEM micrographs shown in Figure 6c,d are in good agreement with the effect in schematic diagram Figure 1c. It is easily found that FG particles have a good contact with each other. Direct contacts among neighboring particles, however, give a higher thermal conductivity enhancement in the composites where particle loading exceeds the critical concentration [27,34]. So it is reasonable to speculate that 40 wt % filling ratio has reached the percolation threshold. Therefore, the interface thermal resistance between FG and HDPE could be effectively suppressed, which is better for heat transport through the composites.

### 3.5. Thermal Stability Analysis about FG/HDPE Composites

**Figure 7:**
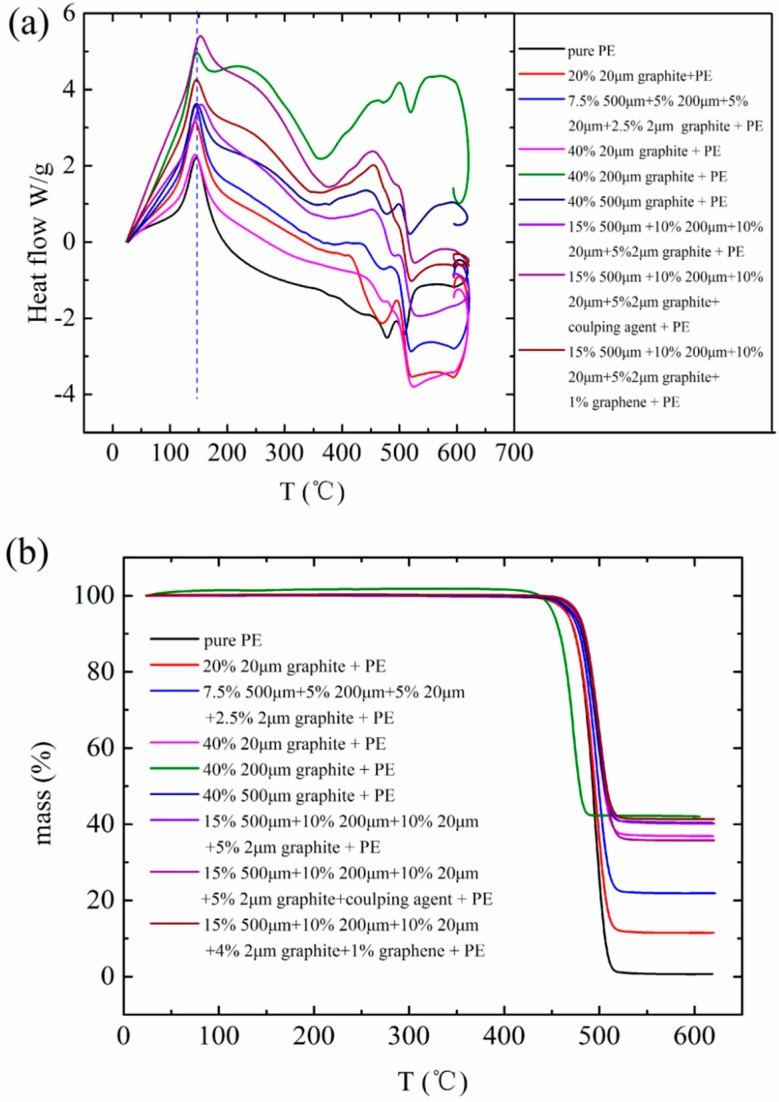
Thermal stability analysis about pure HDPE and relevant composites: (**a**) differential scanning calorimetry (DSC) scans of pure HDPE matrix and HDPE/FG composites and (**b**) thermogravimetric analysis (TGA) of pure HDPE matrix and HDPE/FG composites.

HDPE/FG composites comprise a new generation of multifunctional materials that combine the properties of HDPE and FG. In this part, we focus on the thermal stability analysis of the material. Therefore, we take DSC and TGA into consideration, results are shown in Figure 7. It could be found in Figure 7a that almost all the composites are equipped with the same curing temperature, which is around 145 °C. The curing temperature is consistent with the melting temperature of pure HDPE. That is to say, there is not apparent bonding interaction existing between HDPE and FG. According to the TGA curves in Figure 7b, all composites start to decompose at 450~500 °C. It is just the decomposition temperature of HDPE. In the end, the existing material of composites at above 500 °C is only FG or graphene. The residual mass reveals the original filling ratio of the filler because the testing sample obtained from a small part of the composite. From the results in Figure 7b, we found that when the filling ratio of FG is 40 wt %, the dispersible uniformity of particles is better than 20 wt % filling ratio. That is, homogeneous distribution of filling particles is easier to be realized under a high filling ratio. One reasonable explanation is that when the mass of filler is comparable to the mass of matrix, the mixing effect of the powder mixing method would be better. Above all, the thermal stability of HDPE/FG composites made by the powder mixing method and the melt-extruding process is pretty good. But one point that needs to be emphasized is that the maximum operating temperature of this composite should be under 140 °C.

### 3.6. Material Strength Analysis about FG/HDPE Composites

**Figure 8:**
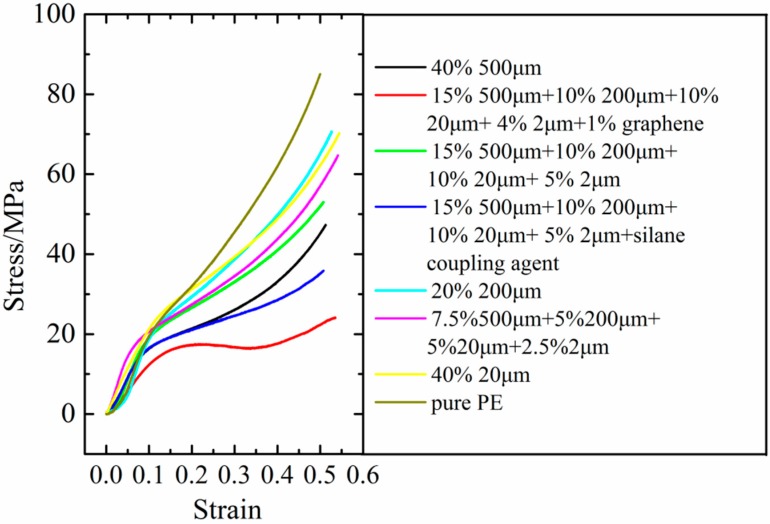
The compression performance curve about the composites.

If one material is prepared to be fabricated in industry, its integrated performance should be excellent. For this consideration, an ordinary compression experiment was employed to evaluate the mechanical strength of these composites in this part. The stress versus compressive strain curves are shown in Figure 8. Results show that all composites have high but lower strength than that of the pure HDPE. And the composites consisted of 20 wt % fillers exhibit better strength than 40 wt % filling ratio. Furthermore, one particle filling pattern possesses higher strength than the several particles filling pattern when the average size of filling particles is smaller than the average size of PE particles. For one particle filling pattern, the stress is higher when the filling particle size is smaller. Because the dispersion uniformity of small fillers is better. Another important finding is that introducing 1 wt % graphene in the fillers is bad for the strength of HDPE/FG composites by comparing the red curve to the green curve. In fact, the large specific surface area of the 1 wt % graphene may hinder the adhesion of HDPE powders, thus the whole cohesive quality of this composite is poor. So we can see that the graphene is not always beneficial to the preparation of composite materials. In conclusion, by combining the thermal conductivity and mechanical strength of these composites, the four kinds of fillers (15 wt % 500 μm/10 wt % 200 μm/10 wt % 20 μm/5 wt % 2 μm FG) are the best choice to produce high thermal conductive HDPE/FG composites.

## 4. Conclusions

In this paper, flake graphite reinforced HDPE composites with a high thermal conductivity were produced by an easy-operating melt-extruding method. According to the results of thermal conductivity measurement, it is found that large fillers play a dominate role in the thermal conductivity when the filling ratio is over 15 wt %. And small fillers own larger specific surface area, so its reinforcing effect and dispersion state are pretty good. By taking full advantage of large FG particles and small FG particles, a special composite (15 wt % 500 μm + 10 wt % 200 μm + 10 wt % 20 μm + 5 wt % 2 μm FG + 60 wt % PE) with a high thermal conductivity about 2.49 W/(m·K) and a good mechanical strength was produced by combining the synergistic effect of several fillers to construct more effective thermal paths. The authors forecast that by further adopting interfacial engineering [35] to control the interface resistance between the PE and graphite, there should be more potential to improve the thermal conductivity of this composite. Then, the SEM image shows the reinforcing effect of small FG particles at the interface of large FG particles. Taking all factors together, such as powder mixing method for better dispersion, twice melt-extruding process to connect each FG fillers and synergistic effect of four FG particles (15 wt % 500 μm + 10 wt % 200 μm + 10 wt % 20 μm + 5 wt % 2 μm FG), a more connecting filler network is formed. DSC and TGA prove the thermal stability of these composites. Consequently, our easy-making flake graphite reinforced HDPE composites with a high thermal conductivity would expand the plastic industrial field of thermally conductive polymers, replacing metals and ceramics in heat transfer devices and equipment, leading to energy and cost savings.

## Figures and Tables

**Table 1:** Average particle size of high density polyethylene (HDPE) matrix and fillers.

Species	HDPE	FG 1	FG 2	FG 3	FG 4	Graphene	MWCNTs
Factory size/μm	250	2	20	200	500	5–10	10–20
Test size/μm	211.27	3.27	19.99	224.05	680.19	5.51	14.32
